# Interfacial Chemistry Involved in Selective Separation of NMC/LMO and LCO/LMO Binary Cathode Materials by Froth Flotation Using Oleic Acid

**DOI:** 10.1021/acsami.5c19071

**Published:** 2026-02-24

**Authors:** Richard K. Oboh, Kaiwu Huang, Seoung-Bum Son, Lei Pan

**Affiliations:** † Department of Chemical Engineering, 3968 Michigan Technological University, Houghton, Michigan 49931, United States; ‡ Department of Mining and Minerals Engineering, Virginia Tech, 445 Old Turner Street, Blacksburg, Virginia 24061, United States; § Chemical Sciences and Engineering Division, 1291 Argonne National Laboratory, Lemont, Illinois 60439, United States

**Keywords:** lithium-ion battery recycling, froth flotation, separation, oleic acid, oleate chemisorption, adsorption

## Abstract

The variability in cathode compositions within recycled lithium-ion battery (LIB) feedstocks poses a significant challenge to efficient downstream refining processes. This study demonstrates the feasibility of using froth flotation with oleic acid as a collector to selectively separate lithium nickel-manganese-cobalt oxide (NMC) and lithium cobalt oxide (LCO) from lithium manganese oxide (LMO) materials. Laboratory-scale flotation tests achieved an 80% separation efficiency in a single stage, producing a froth product with >90% purity of NMC/LCO at approximately 90% yield. Concurrently, the LMO materials were enriched in the sink product with ∼90% purity and ∼90% yield. This approach was further validated using recycled cathode materials, confirming its applicability to realistic feedstocks. The underlying mechanism governing the selective separation of NMC/LCO from LMO was investigated using ζ-potential measurements, contact angle measurements, bubble-particle attachment experiments, and X-ray photoelectron spectroscopy (XPS) analysis. Both contact angle and bubble-particle attachment results confirmed that oleic acid adsorption rendered NMC and LCO surfaces hydrophobic, thereby enhancing flotation recovery. At pH 5, oleic acid adsorbed preferentially onto NMC and LCO surfaces via electrostatic interactions, while exhibiting minimal adsorption on LMO surfaces. However, separation efficiency deteriorated at higher pH, which was attributed to the co-flotation of LMO materials caused by oleate chemisorption on MnOH^+^ species. This work establishes froth flotation as a viable cathode/cathode separation strategy, providing a low-cost, scalable pathway to preconcentrate and enrich nickel-rich and cobalt-rich cathode active materials from incompatible cathode chemistries for direct recycling or hydrometallurgical processing. Furthermore, this study reveals, for the first time, the mechanism of oleate adsorption on the surface of different cathode materials.

## Graphical abstract

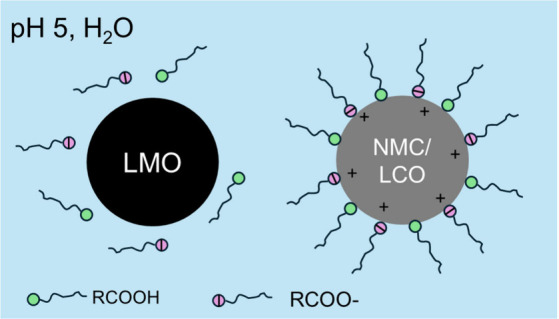

## 1 Introduction

The rising energy demand, particularly the demand for clean energy, has made lithium-ion batteries (LIBs) the dominant energy storage technology for applications ranging from electric vehicles and consumer electronics to renewable energy systems. Modern LIBs offer higher energy density, longer cycling life, and lower cost compared to the earlier battery technologies.^1–3^ As a result, global LIB demand reached 1,100 GWh in 2023 and is expected to increase rapidly to 4,700 GWh by 2030.^4^ Although significant advancements have been made to extend the cycling life of existing LIB technologies, the service life for today’s LIBs remains between 5 and 20 years.^5–7^ The widespread adoption of LIBs has created a major challenge in sustainably managing end-of-life batteries. Improper disposal of LIBs in landfills can cause irreversible environmental damage, including the release of toxic fluorine-containing chemicals.

The development of a circular economy not only mitigates the environmental footprint of battery technologies but also significantly reduces dependence on mining and processing of primary ores.^8–10^ LIBs represent valuable secondary resources for critical materials.^9,11^ A single 60 kWh electric vehicle (EV) battery pack contains more than 100 kg of critical minerals, including lithium, cobalt, nickel, manganese, graphite, aluminum, and others. Recycling LIBs therefore not only eliminates hazards associated with waste materials but also returns valuable raw materials back into the manufacturing supply chain.

Black mass is the fine fraction produced during the shredding and milling of spent LIBs and consists primarily of anode and cathode materials, along with small amounts of aluminum (Al) and copper (Cu). In currently commercialized technologies, such as pyrometallurgical and hydrometallurgical technologies, the economic value of black mass largely depends on its contents of valuable metals such as nickel (Ni) and cobalt (Co), with lesser contributions from lithium (Li) and graphite. In contrast, direct recycling has emerged as a sustainable and cost-effective approach for recovering and reusing materials from lightly degraded cells,^12^ manufacturing scraps,^13^ and end-of-life LIBs.^14^ Rather than extracting metals through energy-intensive pyrometallurgical and hydrometallurgical processes, direct recycling preserves cathode materials through physical and mechanical separation processes, including gravity separation, magnetic separation, and froth flotation.^15–17^ Physically recovered cathode materials can subsequently be repaired via relithiation to restore lithium content.^18,19^ For older cathode chemistries, upcycling strategies may be employed to convert them into newer cathode chemistries with significantly improved electrochemical performance.^20–22^ Compared to pyrometallurgical and hydrometallurgical routes, direct recycling exhibits significant advantages, including (1) lower operating cost, (2) higher product values, and (3) reduced environmental footprint.

However, direct recycling faces significant challenges, especially when dealing with mixed cathode materials. Most existing direct recycling methods are designed for homogenous feedstocks derived from LIBs with single cathode chemistry.^23^ To date, researchers have successfully regenerated single-type cathodes like lithium cobalt oxide (LCO),^24–26^ lithium nickel-manganese-cobalt oxide (NMC),^27–29^ lithium manganese oxide (LMO),^30,31^ and LFP.^32–34^

The presence of multiple cathode chemistries in recycled feedstocks complicates downstream relithiation and regeneration processes.^35^ One strategy to address this challenge is the effective separation of mixed cathode materials into individual components.^36,37^ Selective leaching technology was developed to separate NMC materials from LMO materials by leaching LMO materials using 1.25 M ascorbic acid at a temperature of 70 °C for 10 min.^43^ The resulting LMO leachate was subsequently used as a reagent to upcycle the Ni-rich cathode to NMC532 cathode. However, a key limitation of this approach is that only the Ni-rich cathode is recovered, while the LMO structure is destroyed during leaching and manganese is recovered solely in ionic form.

Froth flotation is a physical separation technique that exploits differences in surface hydrophobicity among particles, using air bubbles as carrier. This technology has been investigated to separate mixed cathode materials. Early studies successfully demonstrated the separation of NMC111 from LMO using a commercial amphoteric collector (Atrac 922).^1^ In a two-stage flotation process, NMC111 with over 95% purity were recovered in the froth product, while LMO materials were concentrated in the sink product. However, the use of proprietary collectors, such as Atrac 922, presents a significant challenge due to its undisclosed chemical compositions. Oboh, Huang, and Pan^38^ demonstrated that sodium octanohydroxamate (OHA) could selectively hydrophobize LCO but not LMO, enabling their separation *via* froth flotation. With sodium metasilicate added as a dispersant to prevent heterocoagulation between LCO and LMO particles, froth flotation achieves an 80% separation efficiency between the two cathode materials in a single flotation stage.

Oleate-based collectors, such as sodium oleate and oleic acid, are widely used in the flotation of oxides, phosphates, as well as rare earth oxides (REOs).^39–41^ These anionic collectors selectively absorb onto mineral surfaces, rendering them hydrophobic.^39,41–43^ Their performance is strongly influenced by pH, temperature, water chemistry, and the presence of modifying agents.^44,45^ Pugh and Stenius^40^ found that sodium oleate forms stable metal–oleate complexes, significantly improving the flotation of apatite, calcite, and fluorite. The flotation efficiency of oleate collectors is strongly pH-dependent, with optimal flotation occurring between pH 8 and 10, where oleate anions dominate, forming surface hydrophobic layers.^44^ In flotation of rare earth oxides, both oleic acid and sodium oleate have achieved over 90% monazite recovery, effectively separating it from hematite and quartz.^46,47^ Dissolved metal species can further influence flotation behavior through surface activation or depression effects.^48^ Acid pretreatment, which removes polyvalent metal ions from mineral surfaces, improves selectivity by reducing unwanted adsorption sites for oleate.^39^ The adsorption of oleate molecules may be categorized into both chemisorption under alkaline conditions and physisorption under acidic conditions. Compared to customized flotation formula, oleic acid exhibits several advantages, including low cost, broad pH flexibility, biodegradability, and intrinsic dispersing properties.^49,50^ Despite these benefits, the effectiveness and selectivity of oleic acid for separating mixed cathode active materials have not been systemically studied.

In this study, a new flotation chemistry based on oleic acid was developed for the separation of mixed cathode materials, including LCO/LMO, NMC111/LMO, NMC622/LMO, and NMC811/LMO. Laboratory-scale froth flotation experiments were conducted to evaluate the separation efficiency between the two cathode materials. The flotation trials were also performed with recycled cathode materials recovered from spent lithium-ion batteries. The underlying separation mechanisms were investigated using ζ-potential measurement, contact angle measurement, bubble-particle attachment measurement, and X-ray photoelectron spectroscopy (XPS).

## 2 Materials and Methods

### 2.1 Materials

Five pristine cathode active materials (CAMs), including lithium cobalt oxide (LiCoO_2_, LCO), lithium manganese oxide (LiMn_2_O_4_, LMO), and lithium nickel manganese cobalt oxide (NMC), i.e., LiNi_0.33_Mn_0.33_Co_0.33_O_2_ (NMC111), LiNi_0.6_Mn_0.2_Co_0.2_O_2_ (NMC622), and LiNi_0.8_Mn_0.1_Co_0.1_O_2_ (NMC811), were used in this work. They were obtained from Toda America Inc. Oleic acid (OA) was obtained from Thermo Scientific (90% purity) and was used as received. Methyl isobutyl carbinol (MIBC, Thermo Scientific) was used as a frother. Sulfuric acid (H_2_SO_4_) was obtained from Sigma-Aldrich. Hydrochloric acid (HCl, Fisher Chemicals) and sodium hydroxide (NaOH, Macron Fine Chemicals) were used to adjust the solution pH. Deionized (DI) water was obtained from a laboratory water purification system (Thermo Scientific). Isopropanol alcohol (IPA) was obtained from VWR chemicals.

### 2.2 Production of Recycled Cathode Materials

Recycled LCO materials were obtained from spent consumer electronics LIB cells, while recycled NMC111 cathode material was obtained from spent prismatic LIB cells. The spent batteries were discharged at a rate of C/10 to 2.8 V and then held at 2.8 V for 24 h to minimize both the fire and explosion risks. After discharging, the spent LIBs were manually disassembled in a fume hood to separate the electrode sheets, including cathode sheets, anode sheets, separators, and casing. The cathode sheets were cut into 1 in. × 1 in. squares and then soaked in 99% isopropanol alcohol (IPA) solution overnight at room temperature (25 °C) to ensure removal of electrolyte and some PVDF binder. The cleaned cathode sheets were rinsed with deionized water and vacuum-dried in an oven at 95 °C for 12 h to remove any residual organics. The dried cathode sheets were fed into a blender with DI water, where the cathode active materials were delaminated from current collectors. The fine cathode active materials in the slurry were then separated from coarse aluminum flakes using a 100-mesh screen sieve.^38^ The undersized product was filtered and dried to obtain the recycled cathode powder, which was used as a feed material for flotation testing.

### 2.3 Laboratory Flotation Trials

Laboratory flotation tests were performed using a 1 L Denver flotation cell. The impeller speed was maintained at 1,350 rpm for all experiments. Aeration was provided by fully opening the cell’s air inlet valve, allowing atmospheric air to enter passively. The froth height was maintained at a constant of 1 cm for all of the tests.

Initially, individual cathode materials (LCO, LMO, NMC111, NMC622, and NMC811) were tested to evaluate their floatability across different pH levels and collector dosages. In each test, 5 g of a given cathode material was mixed with 1 L of DI water to form a pulp. The pulp pH was controlled using HCl or NaOH. Once desired pH was reached, oleic acid (OA) was added as a collector and conditioned for 5 min, followed by the addition of methyl isobutyl carbinol (MIBC) as frother for another 2 min. After conditioning, air was introduced, and froth products were collected at 0.5, 1, 2, and 3 min to determine flotation kinetics. The total flotation time for each test was 3 min.

Binary separation tests of mixed cathode materials, i.e., LCO/LMO, NMC111/LMO, NMC622/LMO, and NMC811/LMO, were also performed using a 1 L Denver flotation cell. 5 g of each cathode was mixed at 1:1 ratio and added to the flotation cell. The mixed feed materials were subjected to conditioning and subsequent flotation trials. The effects of the pH and collector dosage were investigated. Samples after the froth flotation trials were assayed using an X-ray fluorescence (XRF) analyzer (EX-6600) and an inductively coupled plasma optical emission spectroscopy analyzer (Agilent 5800 ICP-OES). From the elemental composition data, both flotation recoveries and product grades were determined. Figure 1 shows the flotation test procedure for separating mixed cathode materials.

**Figure 1:**
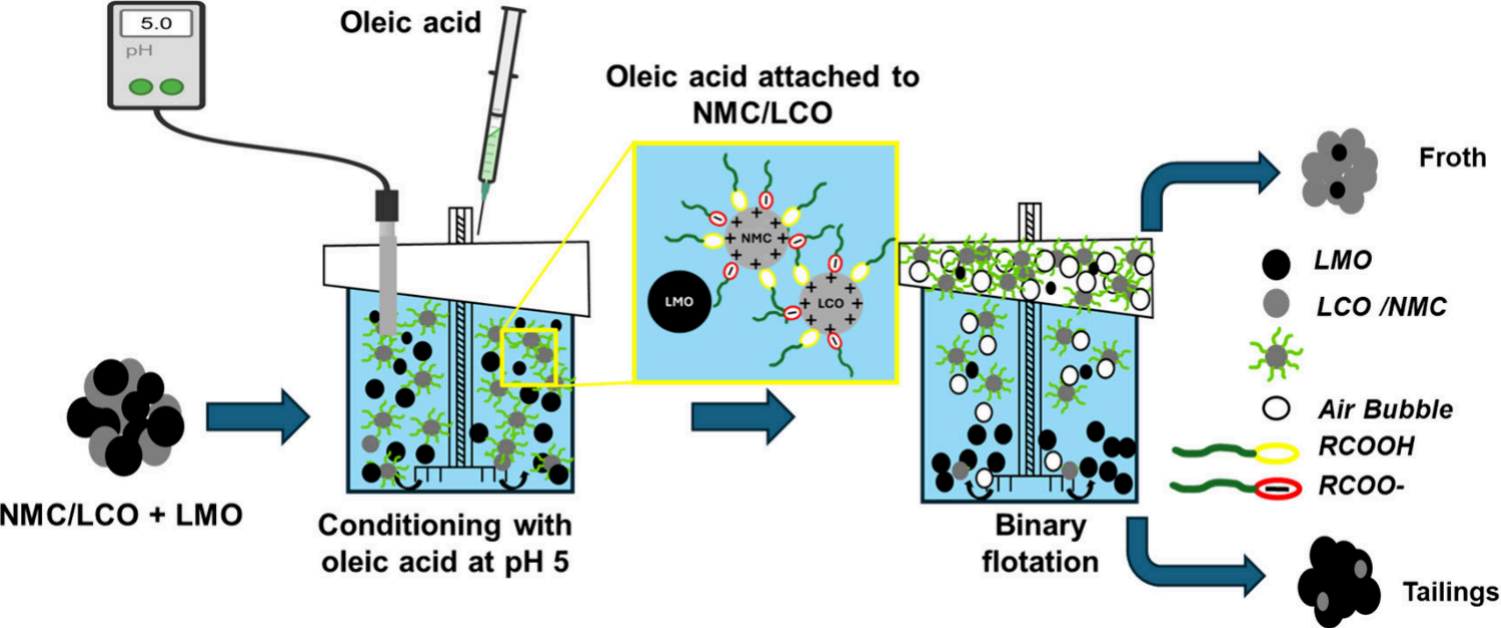
Procedure of flotation experiment to separate mixed cathode materials.

### 2.4 Characterization

Both the bulk and surface properties of the cathode active materials were characterized. Particle size distribution (PSD) of the cathode materials was determined by using a particle size analyzer (MicroTrac Sync). The ζ-potentials of all cathode materials were determined by using a Malvern Zetasizer (NanoZS, ZEN3600). The ζ-potential measurements were performed using the submicrometer fraction of the ground cathode materials. Ultrafine grinding was performed using a laboratory attrition mill (Union Process, 01HDT) with 4 mm ceramic spheres prior to the ζ-potential measurement. The ground materials were transferred into a beaker and allowed to free settle for 10 min. The suspended particles in the slurry were then used in the ζ-potential measurement. 10^–5^ M sodium chloride (NaCl) was used as a background electrolyte during each measurement. Five measurements were performed, and the results were averaged.

Composition as well as morphology of the separated products were analyzed using a Field Emission Scanning Electron Microscope (Apreo 2 FE-SEM, Thermo Fisher) equipped with energy-dispersive X-ray spectroscopy (EDS). Elemental mapping was also performed to reveal the distribution of different cathode materials in the separated product.

X-ray photoelectron spectroscopy (XPS) analysis was performed using a PHI 5800 spectrometer (Physical Electronics), with a 100 W Mg Kα X-ray (1253.6 eV) as the energy source. The spectra were acquired in both survey and high-resolution modes. For the survey scans, the instrument was operated at a pass energy of 187.85 eV, a data spacing of 0.8 eV, and a dwell time of 20 ms. High-resolution spectra were collected by using a pass energy of 23.50 eV, data spacing of 0.1 eV, and dwell time of 100 ms. A charge neutralizer was employed during the analysis to correct for surface charging effects. The acquired spectra were fitted and analyzed by using Multipak software.

Contact angle measurements were performed to determine the surface hydrophobicity (wettability) of various cathode materials. Two types of cathode samples were prepared, i.e., cathode samples conditioned in deionized water and the samples conditioned with 400 g/t oleic acid at pH 5. The conditioning was conducted in a 1 L Denver flotation cell. After conditioning, samples were filtered, rinsed thoroughly with deionized water, and left to air-dry for 24 h. The dried powder was then gently spread and pressed onto a clean glass slice to form a uniform layer approximately 1 mm thick. The cathode-coated glass slice was loaded to a Rame-Hart goniometer for contact angle measurement using the sessile drop method, in which small droplets of deionized water were placed onto the sample surface. The droplet profile was captured using a high-resolution camera, and the contact angle was calculated using image analysis software. Multiple measurements were recorded for each sample to ensure repeatability.

Bubble-particle attachment study was conducted using the setup shown in Figure 2. This setup enabled a direct visualization of the role of oleic acid on the bubble-particle attachment process, which was critical in froth flotation. The procedure of preparing cathode samples was the same as the one used for contact angle measurement. The prepared cathode powders were added to the bottom of a glass cell filled with deionized water. At the same time, a single air bubble (1–3 mm in diameter) was generated using a small capillary tube over the cathode powders in water. The bubble-particle attachment experiment was initiated by lifting the glass cell using a micromanipulator so that the cathode particles were approaching the suspended air bubble slowly. When the particles were contacting the bubble surface, the approaching was stopped and the glass cell started retracting. During this approaching and retracting process, a high-speed side camera was used to capture whether the particles attached, detached, or failed to interact with the suspended bubble.

**Figure 2:**
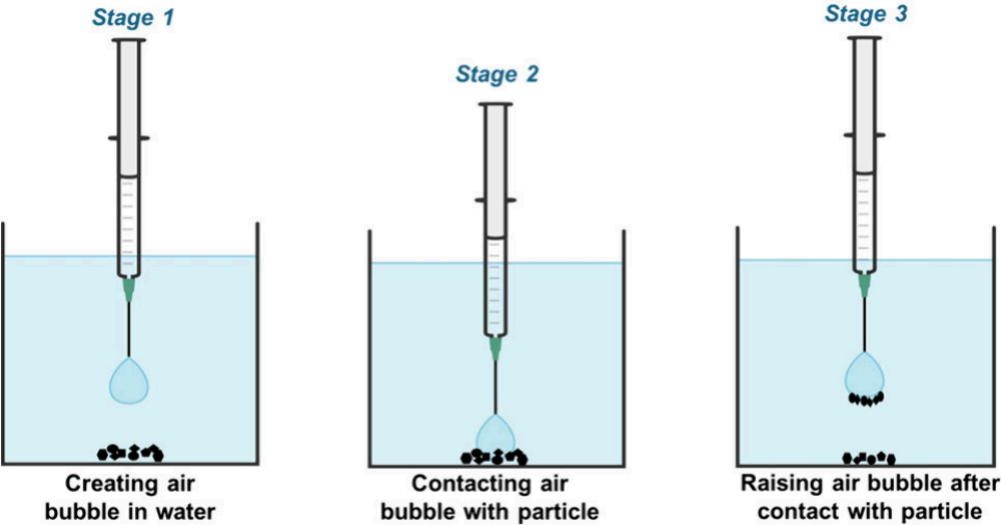
Laboratory setup for bubble-particle attachment studies.

## 3 Results

### 3.1 Feed Characterization

Table 1 shows the elemental compositions of various pristine cathode active materials (CAMs). The elemental composition was determined using ICP-OES. Lithium content for these materials ranged between 4.1% and 7.1%. Note also that lithium content within NMC materials increases with increasing nickel content within the NMC materials.

**Table 1:** Elemental Composition of Pristine Cathode Active Materials

CAM	Li (%)	Ni (%)	Mn (%)	Co (%)
LCO	7.1	0.0	0.0	63.1
LMO	4.1	0.0	57.8	0.0
NMC111	6.5	18.2	15.7	17.8
NMC622	7.3	43.9	12.2	12.5
NMC811	6.1	41.8	4.9	5.1

Figure 3 shows the particle size distribution (PSD) and scanning electron microscopy (SEM) images of five different cathode materials, including NMC111, NMC622, NMC811, LMO, and LCO. Considerable variation in the median particle size was observed among these materials. The NMC111 sample exhibited the finest particle size, with a medium particle size (D50) of 9 μm and 80% passing size (D80) of 12 μm. NMC622 and NMC811 showed slightly larger particle sizes, with D50 values of 14 and 15 μm and correspondingly D80 values of 18 and 21 μm, respectively. Both the LMO particles and LCO particles are slightly coarser than NMC materials. The D50 and D80 sizes for the LMO materials are 13 and 21 μm, respectively, and the D50 and D80 sizes for the LCO materials are 17 and 25 μm.

**Figure 3:**
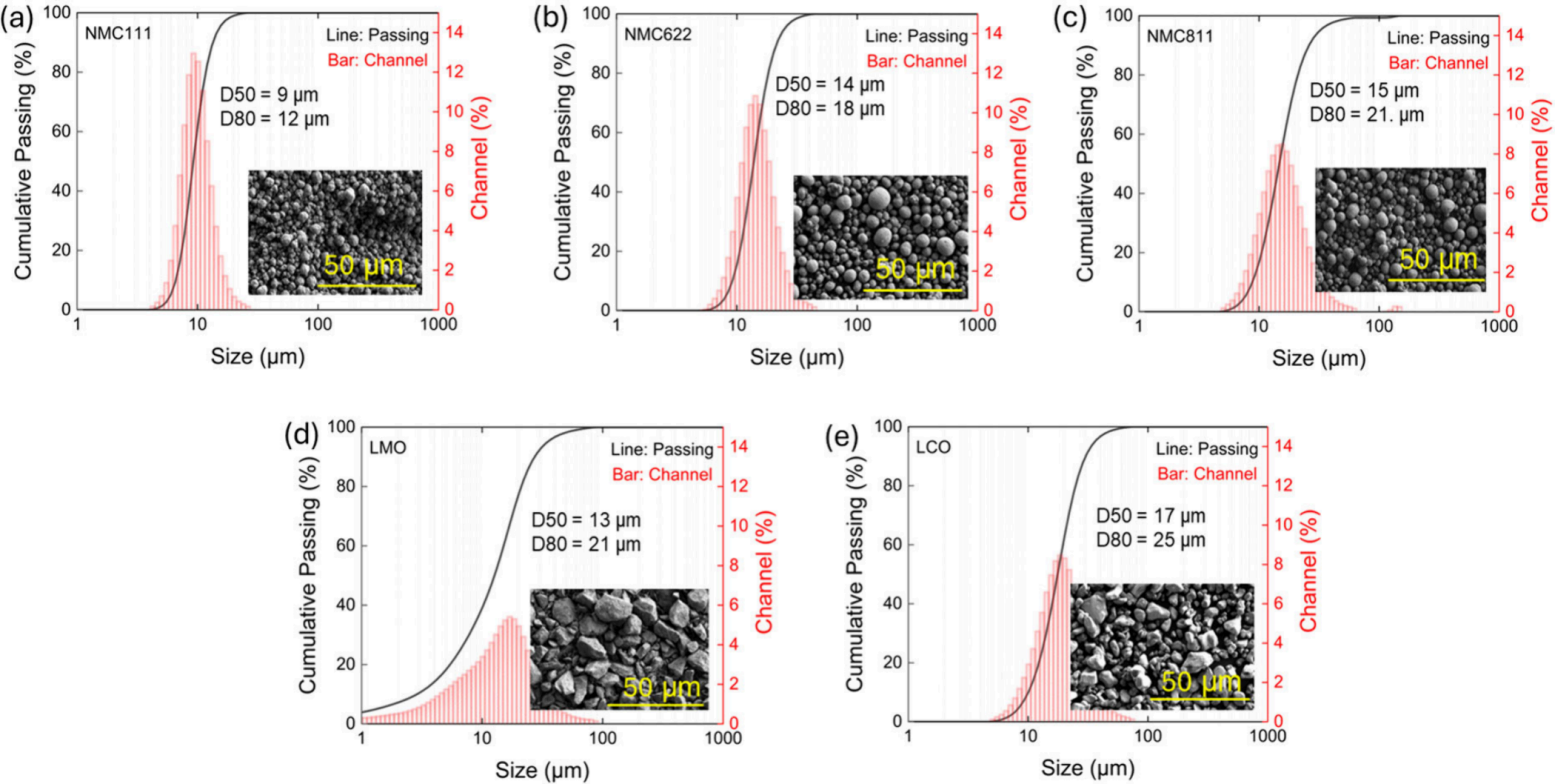
Particle size distribution and SEM images of a) NMC111, b) NMC622, c) NMC811, d) LMO, and e) LCO cathode active materials.

The SEM images in Figure 3 present the morphological differences among the cathode materials. The NMC-based materials (NMC111, NMC622, and NMC811) appeared as spherical and uniform particles. In contrast, both the LCO and LMO exhibit morphologies with distinct surface textures. LCO particles display comparatively smooth surfaces, whereas LMO particles exhibit rough, highly fragmented surfaces with angular features.

### 3.2 Flotation of Individual Cathode Active Materials

#### 3.2.1 Effect of pH and Collector Dosage

Flotation tests were conducted by using individual cathode materials as the feed. Oleic acid (OA) was used as a collector. Figure 4 presents the flotation results of individual cathode materials across varying pH conditions (5–11) and collector dosages (100–400 g/t). The results show that the recovery of individual cathode materials depends on both the solution pH and collector dosage. NMC and LCO cathodes exhibited the highest recoveries at pH 5. At this condition, over 95% flotation recoveries were achieved for NMC111, NMC622, NMC811, and LCO. However, increasing the pH from 5 to 9 led to a gradual decrease in flotation recoveries and a significant drop at pH beyond 9. The reduced recovery of LCO and NMC materials under strongly alkaline conditions (pH > 9) may be attributed to surface hydroxylation and the formation of stable metal hydroxides (e.g., Co­(OH)_2_, Ni­(OH)_2_).^51^ These hydroxide layers passivate the surface, which hindered collector adsorption and consequently lowered the surface hydrophobicity.^43^

**Figure 4:**
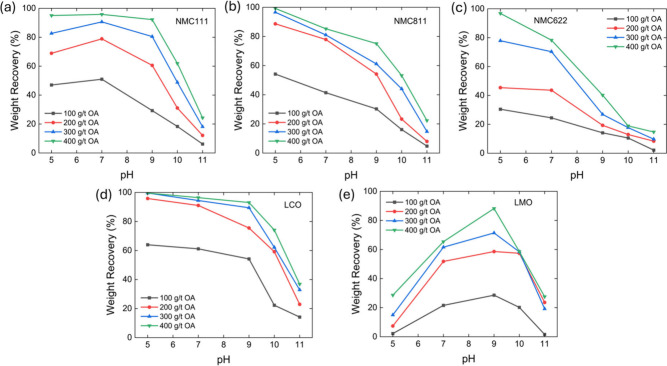
Flotation recovery of various cathode materials with different oleic acid dosages as a function of pH. (a) NMC111, (b) NMC811, (c) NMC622, (d) LCO, and (e) LMO.

The flotation behavior of LMO differs significantly from that of NMC and LCO. As shown in Figure 4e, LMO recovery increased with rising pH across all collector dosage ranges when the pH was below 9. However, when the pH was further increased from 9 to 11, LMO recovery dropped to below 20%. This observation is consistent with the earlier finding for the flotation of manganese dioxide (MnO_2_),^45^ which showed over 90% flotation yield for manganese dioxide (MnO_2_) at pH 8–10 using OA as a collector but minimum flotation recovery at pH 5–6.

The high flotation recovery of LMO at pH 9 can be explained by the unique pH-dependent hydrolysis of manganese ions. In the near-neutral to mildly alkaline range, the dominant surface species is likely positively charged MnOH^+^. This species creates a favorable electrostatic attraction for the anionic oleate collector (RCOO^–^), enhancing its adsorption and leading to increased flotation recovery.^45,52^ However, at higher pH (>10), further hydroxylation results in the precipitation of neutral Mn­(OH)_2_, which may suppress collector adsorption and reduce floatability.

### 3.3 Separation of Mixed Pristine Cathodes

The results obtained with individual cathode materials indicate that oleic acid is a promising collector candidate for selectively separating LMO from LCO and NMC cathodes. To validate this hypothesis, laboratory flotation trials were performed using cathode mixtures of LCO/LMO, NMC111/LMO, NMC622/LMO, and NMC811/LMO at 1:1 ratio by weight. The mixtures were subjected to flotation at pH 5.0 at varying oleic acid dosages of 200, 300, and 400 g/t. These tests aimed to evaluate the selectivity and effectiveness of oleic acid in separating LMO from LCO and NMC-type cathodes.

Figure 5 shows the flotation results obtained with a mixture of NMC111 and LMO cathode materials at OA dosages of 200, 300, and 400 g/t. Cumulative recovery is shown as a function of flotation time. Figure 5a shows that NMC111 exhibited an excellent flotation response at pH 5.0, reaching 68% within 3 min of flotation. When the dosage was increased to 400 g/t, the NMC111 yield reached 90%. In contrast, LMO exhibited a poor flotation response to flotation performance across all three dosages, as shown in Figure 5b. Even at a dosage of 400 g/t, LMO recovery remained below 10% after 3 min.

**Figure 5:**
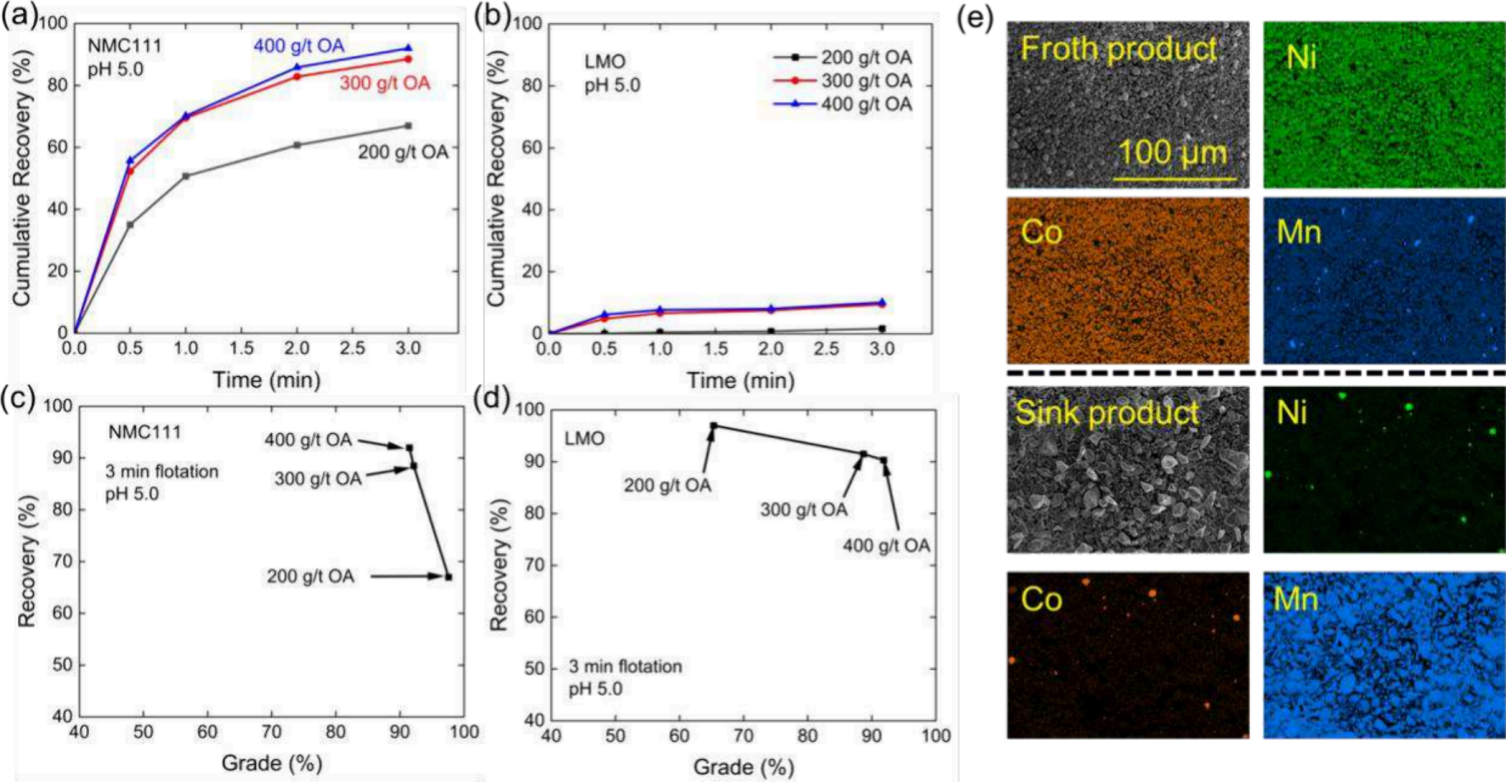
(a) Cumulative recovery of NMC111 at pH 5.0 under varying oleic acid (OA) dosages (200, 300, and 400 g/t) in binary flotation experiments, (b) cumulative recovery of LMO at pH 5.0, (c) recovery *vs* grade curve with NMC111 in the froth product at pH 5.0 using different oleic acid (OA) dosages, (d) recovery *vs* grade curve with LMO in the tailing product at pH 5.0 using different oleic acid (OA) dosages, and (e) SEM images and elemental mapping of both the froth and sink products.

Figure 5c shows the recovery *vs* grade curve for NMC111 in the binary separation between NMC111 and LMO. The three data points were obtained at varied OA dosage. As shown, increasing OA dosage from 200 to 400 g/t significantly improved the recovery of NMC111, while the grade of NMC product was slightly compromised due to introduction of LMO materials in the froth product. Figure 5d shows the recovery *vs* grade curve of LMO in the sink product. With an increase in OA dosage, more LMO became floatable and consequently lowered the overall recovery of the LMO materials in the sink product. At 400 g/t OA doasge, over 90% grade was achieved for both NMC111 and LMO products. Figure 5e shows SEM images and elemental mappings of both the froth and sink products, providing direct visual evidence that NMC and LMO could be effectively separated by flotation using OA as a collector.

Separation efficiency (SE) is a commonly used metric to quantify the effectiveness of a separation process. For a binary separation system, SE can be determined using the following equation^53^

1 $$SE=\left|R_{1}-R_{2}\right|$$

where *R*_1_ is the recovery of one component in the concentrate product and *R*_2_ is the recovery of the other component in the concentrate product. A higher SE value indicates more effective separation between the two cathode materials.

A series of flotation tests were performed to evaluate the separation efficiency with other binary cathode mixtures including NMC622/LMO, NMC811/LMO, and LCO/LMO. Oleic acid (OA) was used as the collector. Figure 6a summarizes the SE values for binary flotation of NMC111/LMO, NMC622/LMO, NMC811/LMO, and LCO/LMO at pH 5.0 and different OA dosages. More detailed flotation results are available in the Supporting Information. For NMC-type cathodes (e.g., NMC111, NMC622, and NMC811), the SE increased with increasing OA dosages, reaching a maximum of ∼85% at 400 g/t OA dosage after one single stage of froth flotation. The increase in the SE was attributed to enhanced surface hydrophobicity of NMC materials with increasing collector dosage. In contrast, the binary separation of the LCO/LMO mixture exhibited a slightly different trend. The SE increased with OA dosage up to 300 g/t but declined to 75% at 400 g/t OA.

**Figure 6:**
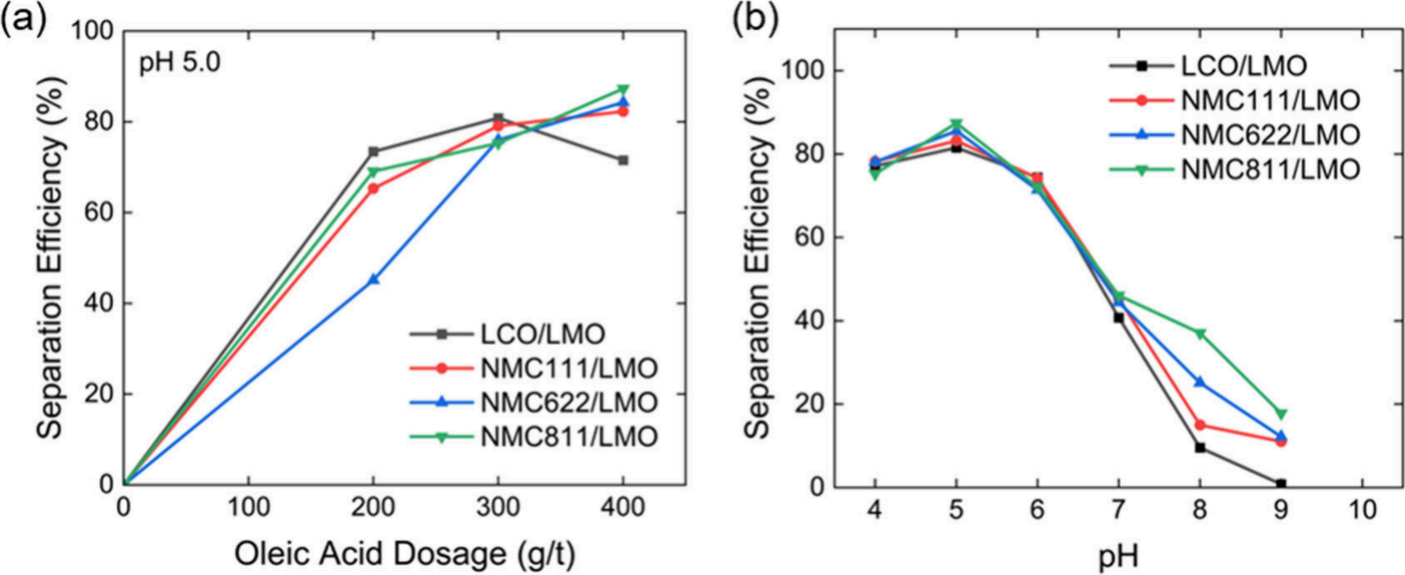
(a) Effect of oleic acid dosage on the separation efficiency (SE) for different binary separation systems. (b) Effect of pH on the separation efficiency (SE) for different binary separation systems.

The impact of solution pH on the separation efficiency (SE) was also investigated, with the results presented in Figure 6b. The optimum oleic acid (OA) dosages were applied for each system, i.e., 400 g/t for NMC cathodes and 300 g/t for the LCO cathode. Across all binary separations, SE followed a consistent trend: maximum efficiency was obtained at pH 5, after which SE declined with increasing pH. This trend indicates that the flotation selectivity of NMC and LCO over LMO is strongly influenced by pH. At a higher pH, more LMO was recovered in the froth, while both LCO and NMC recoveries decreased, leading to a decrease in separation efficiency.

Figure 7 shows a summary of the flotation results with various cathode mixtures. The recovery of LMO (*R*_1_) is plotted vs the recovery of the other cathode material (*R*_2_), with reference separation efficiency (SE) lines shown in the diagonal direction. As shown, SE for all binary separation systems was found to be over 80%, confirming an excellent separation performance. Correspondingly, the recovery rates of NMC811, NMC622, NMC111, and LCO in the froth product were 90% for all binary separation systems. High-grade cathode products were obtained at high flotation recoveries. These results highlight the effectiveness of oleic acid in selectively separating different NMC-type cathodes (NMC111, NMC622, and NMC811) and LCO from LMO.

**Figure 7:**
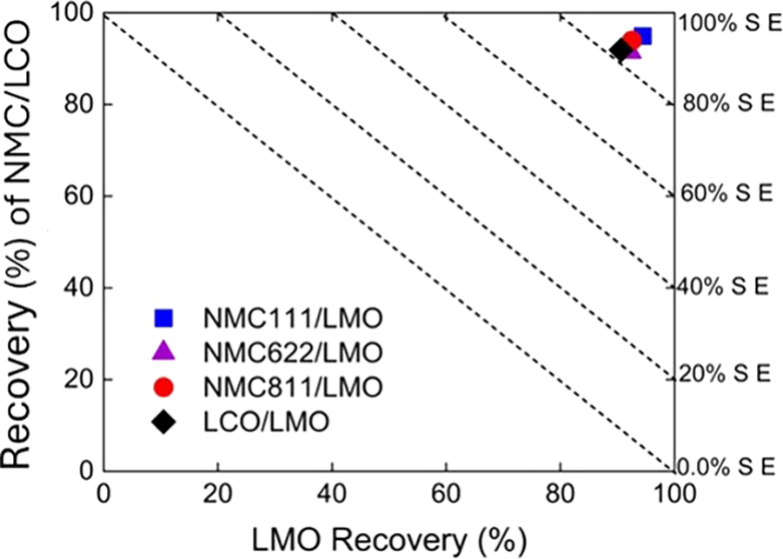
Recovery of NMC and LCO *vs* recovery of LMO at pH 5.0 using oleic acid as a collector. The dashed lines in the diagonal direction represent the separation efficiency (SE).

### 3.4 Binary Separation of Recycled Cathode Materials

The aim of this part is to evaluate whether the flotation behavior of spent cathode materials differs from that of pristine cathodes. Table 2 shows the elemental composition of the two recycled cathode active materials, determined by ICP analysis. The elemental compositions of recycled LCO and NMC111 materials are very similar to those of the pristine cathode active materials. The slight reduction in lithium content (∼0.6% absolute) is characteristic of spent materials. This is primarily due to the irreversible retention of lithium in the graphite anode during cycling,^54^ with minor contributions from potential dissolution during the washing and mechanical delamination steps. Note also that trace amounts of impurities, such as copper and aluminum, were also detected in the recycled cathode active materials.

**Table 2:** Elemental Composition of Recycled Cathode Active Materials

CAM	Li (%)	Ni (%)	Mn (%)	Co (%)	Al (%)	Cu (%)
Recycled LCO	6.47	0.0	0.0	58.22	0.09	0.02
Recycled NMC111	6.51	17.10	14.13	16.18	0.21	0.01

Flotation tests were performed to separate pristine LMO materials from recycled cathode materials (LCO and NMC111) obtained from spent lithium-ion batteries. The tests were carried out at pH 5.0 using oleic acid (OA) as the collector, with the results shown in Figure 8. Figure 8a illustrates the stepwise procedure of recovering recycled NMC111 and LCO cathodes as well as the separation of the recycled cathode after it is mixed with pristine LMO. As shown in Figure 8a, spent battery was shredded and delaminated. Cu, Al, and plastics were separated from black mass. The cathode materials was separated from the black mass using the anode cathode separation method adopted from refs 15 and 55 .

**Figure 8:**
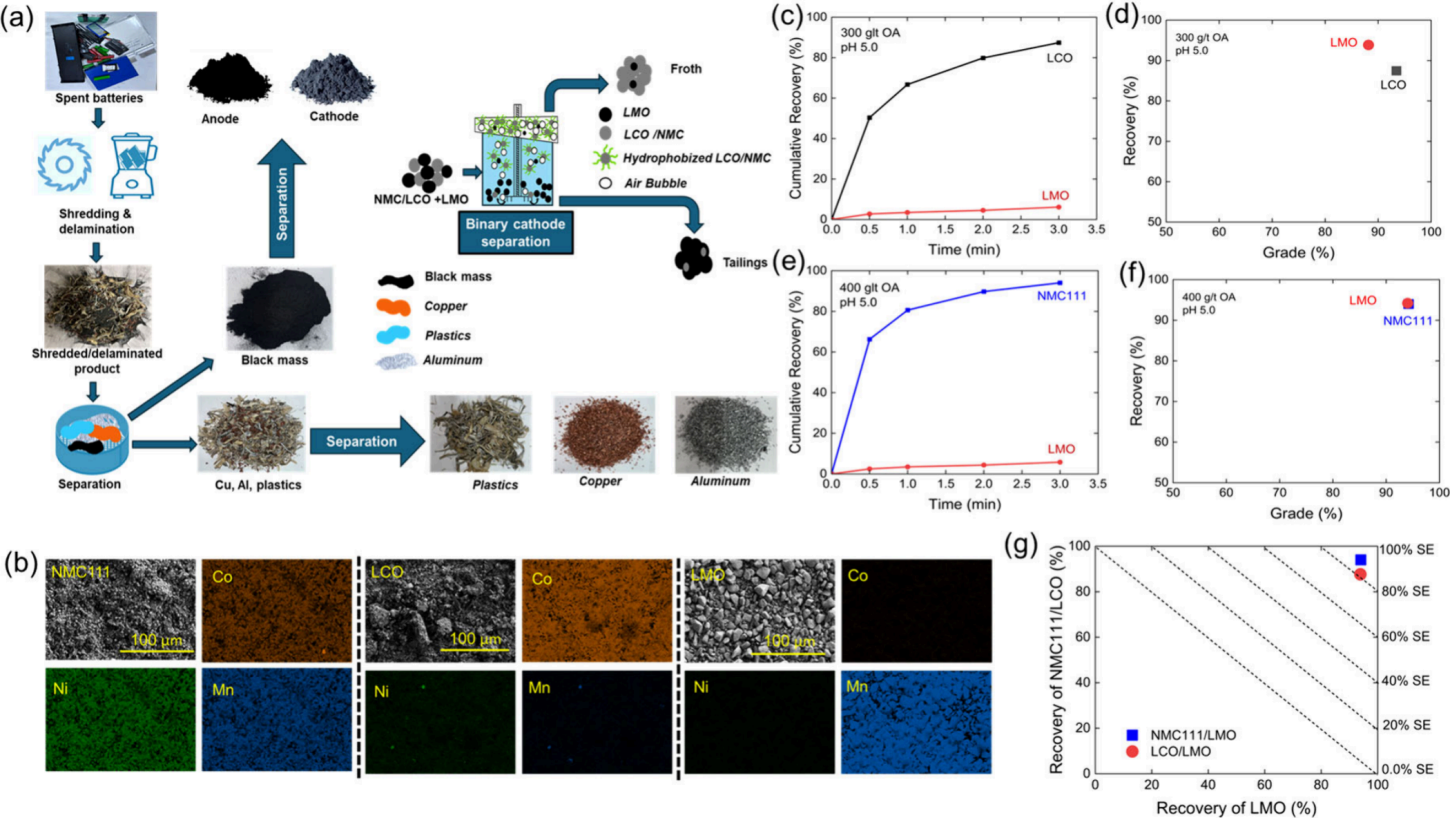
(a) Stepwise procedure for recycled cathode recovery and separation. (b) SEM and elemental mapping images of recycled NMC111, recycled LCO, and pristine LMO. (c–e) Flotation recoveries of LCO/LMO and NMC111/LMO in the froth product at pH 5.0 using oleic acid as the collector (300 g/t for LCO/LMO and 400 g/t for NMC111/LMO separations). (d–f) Recovery *vs* grade curves for LCO/LMO and NMC111/LMO separations and (g) separation efficiency plots for NMC111/LMO and LCO/LMO separations.

Figure 8c shows the flotation results obtained with a binary mixture of recycled LCO and pristine LMO materials. As shown, over 80% of recycled LCO materials were floated within 3 min of flotation at 300 g/t of OA and pH 5, while the majority of LMO cannot be floated. The recovery *vs* grade curve is presented in Figure 8d. The froth product contained more than 90% LCO, with an LCO recovery of over 85% in the froth product. Conversely, the LMO recovery in the sink product exceeded 90% with nearly 90% purity.

Figure 8e and 8f shows the results obtained between the recycled NMC111 materials and the pristine LMO materials. The recycled NMC111 exhibited strong floatability, reaching over 90% recovery within 3 min of flotation, while the majority of LMO materials remained in the pulp phase. This behavior is consistent with the results obtained with the recycled LCO materials. The results demonstrate that spent cathodes can be efficiently separated using the froth flotation method with oleic acid as a collector. Figure 8g shows the recoveries of recycled NMC and LCO materials *vs* recovery of LMO obtained at pH 5.0, with reference separation efficiency (SE) lines shown in the diagonal direction. As shown, the separation efficiencies for LCO/LMO and NMC111/LMO separation were both over 80%.

## 4 Discussion

### 4.1 Wettability

To better understand the flotation response of individual cathode materials, contact angle measurements were performed using the sessile drop method. The cathode materials were conditioned with OA at pH 5, and powder-coated surfaces were prepared as described in Section 2.4. Figure 9 presents side-view images of sessile water droplets on cathode samples treated with oleic acid at pH 5. The reported contact angles are the average of five replicate measurements per sample (each water droplet in the inset at the top-left corner represents one measurement). The OA-treated LCO surface exhibited the highest contact angle of 145° as shown in Figure 9c. OA-treated NMC111 materials also exhibited some degree of hydrophobicity, with a contact angle of 86° as shown in Figure 9b. The hydrophobicity of the LCO and NMC111 materials is consistent with their floatability in the flotation trials. In contrast, the water droplet completely spread and wetted the OA-coated LMO surface in all five measurements (Figure 9a), confirming its hydrophilic nature even after conditioning.

**Figure 9:**
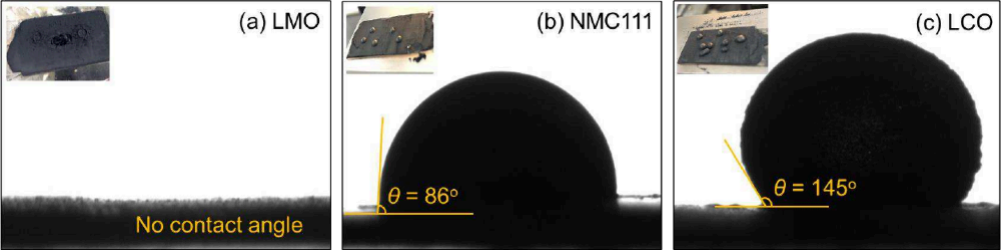
Contact angle measurement of varying battery feed materials after oleic acid conditioning at pH 5: (a) LMO, (b) NMC111, and (c) LCO.

Bubble-particle attachment behavior of individual cathode materials was also examined using the customized bubble-particle attachment setup shown in Figure 2. Samples were tested with and without oleic acid (OA) conditioning at pH 5. Figure 10 shows images of air bubbles after being in contact with particle beds with and without OA conditioning. In the absence of OA, air bubbles picked up a small amount of cathode materials due to their weak surface hydrophobicity. After OA conditioning, however, both LCO and NMC111 particles were picked up by air bubbles, suggesting that LCO and NMC111 became hydrophobic after OA conditioning. On the other hand, a limited amount of LMO particles were picked up by the air bubble after OA conditioning, suggesting that OA cannot increase the hydrophobicity of LMO particles. The results shown in Figure 10 further confirm that OA conditioning at pH 5 effectively renders LCO and NMC111 surfaces hydrophobic, while it has little effect on the surface hydrophobicity of LMO.

**Figure 10:**
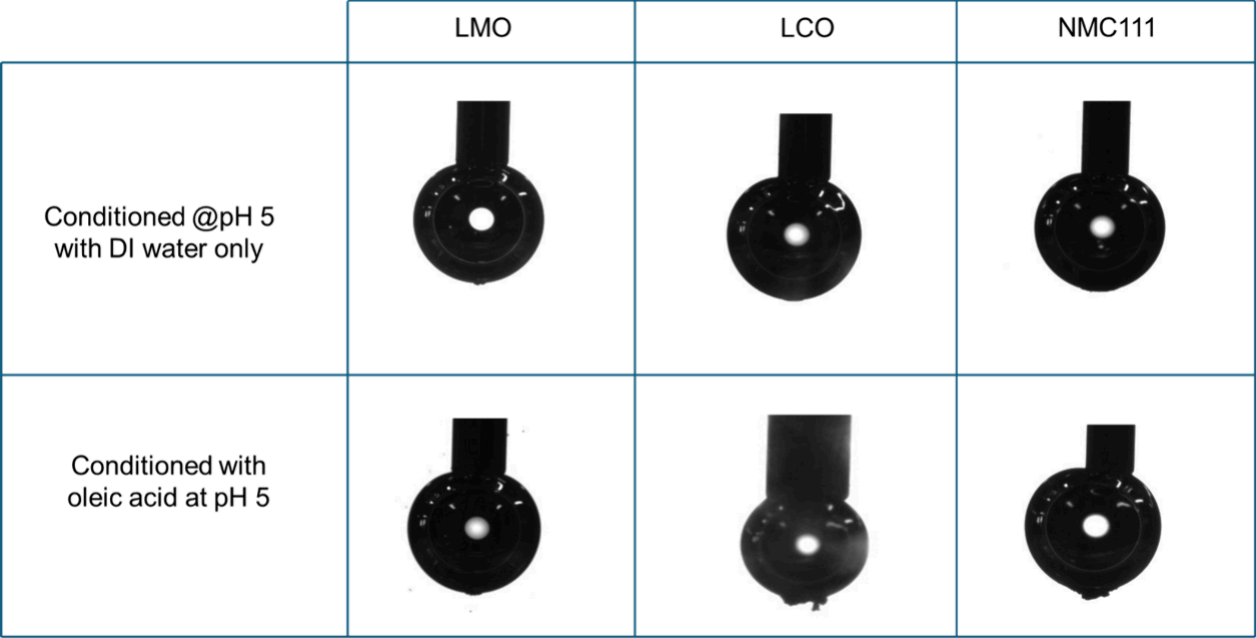
Images of air bubbles obtained in the bubble-particle attachment measurement.

### 4.2 XPS Analysis

To gain insight into the adsorption mechanism of OA on cathode surfaces, XPS analyses were performed using cathode materials with and without oleic acid (OA) conditioning. Figure 11 presents the high-resolution O 1s spectra for LCO and LMO materials before and after conditioning with OA. The hypothesis is that OA preferentially adsorbed on the LCO surface rather than the LMO materials after OA treatment at pH 5.0. Each spectrum was deconvoluted into chemical components including metal oxides (metal–O) at 529.05 eV and hydroxide (−OH) at 529.86 eV, the C=O bond at 531.45 eV, and the C–O bond at 532.56 eV. Figure 11a shows the deconvoluted O 1s spectrum obtained with the untreated LCO surface, whereas Figure 11b shows the O 1s spectrum obtained with the LCO materials after OA treatment. Table 3 compares the percentage by weight of each characteristic oxygen bond on LCO surfaces before and after OA treatment.

**Figure 11:**
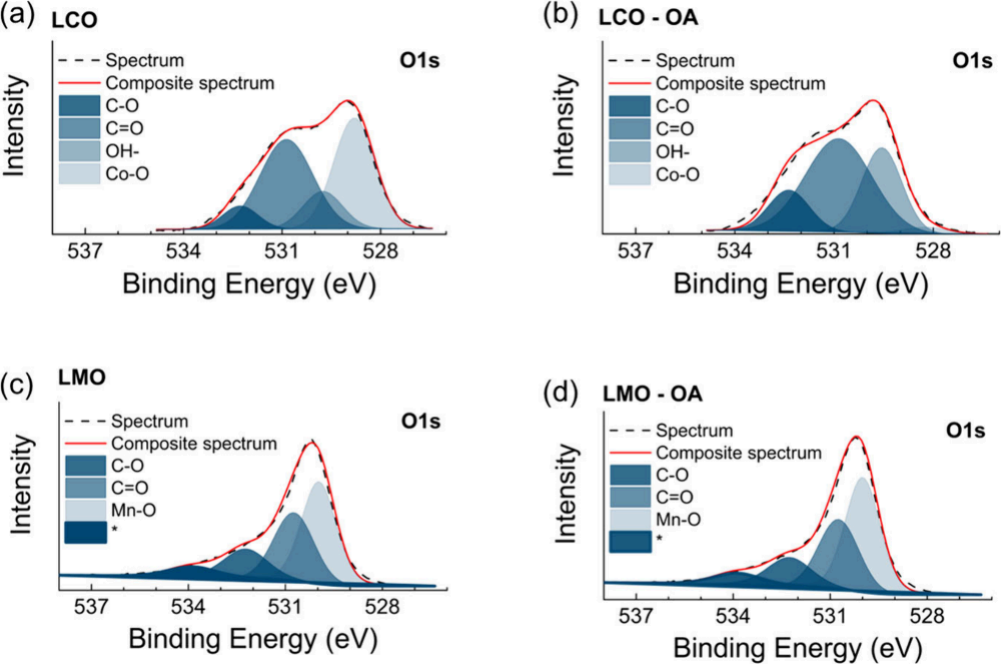
High-resolution XPS O 1s spectra of (a) LCO feed conditioned at pH 5, (b) LCO after oleic acid treatment at pH 5.0, (c) LMO feed conditioned at pH 5, and (d) LMO after oleic acid treatment at pH 5.0.

**Table 3:** O 1s Spectrum Analysis of LCO and LMO Materials before and after Oleic Acid Conditioning

	LCO	LCO-OA	LMO	LMO-OA
Metal–O	56.16%	6.69%	70.08%	71.25%
OH	10.7%	51.31%	5.09%	6.24%
C=O	27.1%	28.67%	12.49%	9.78%
C–O	6.05%	13.33%	12.34%	12.73%

As shown, the LCO surface was dominated by the metal oxide bond. As shown, the metal–O bond contributed to the 56.16% peak area, whereas the −OH, C=O, and C–O bonds account for 10.7%, 27.1%, and 6.05%, respectively. After the treatment with OA, the metal–O peak decreased substantially to 6.69%, while −OH became the major peak contributing to the 51.31% peak area. The peak areas for C=O and C–O also increased to 28.67% and 13.33%, respectively. The decrease in the metal–O peak and increase in C=O and C–O are likely due to the adsorption of oleic acid on the LCO surface. The adsorption of oleic acid is responsible for the enhanced surface hydrophobicity of LCO and, hence, its floatability.

On the other hand, the O 1s spectra for LMO before and after oleic acid conditioning at pH 5 did not show significant differences, as shown in Figure 11c and d, suggesting that OA did not adsorb on the LMO surface, which led to its low hydrophobicity. The XPS spectra further confirm that oleic acid can selectively make LCO hydrophobic by adsorption while not reacting with LMO. Due to its high selectivity, oleic acid is a promising collector for separating LMO cathodes from others. Further insights could be gained from high-resolution spectra metal-edge analysis of Ni and Co.

### 4.3 ζ-Potential Analysis

In the flotation of oxide minerals, an electrical double layer at the mineral–water interface governs collector adsorption (Fuerstenau and Pradip, 2005). The change in the ζ-potential before and after collector adsorption provides insight into the adsorption mechanism. Figure 12 shows the ζ-potentials of five individual cathode materials (LCO, LMO, NMC111, NMC622, and NMC811) at different pH values in the presence of varying oleic acid (OA) concentrations. A background electrolyte of 1 × 10^–5^ M NaCl was used for all measurements.

**Figure 12:**
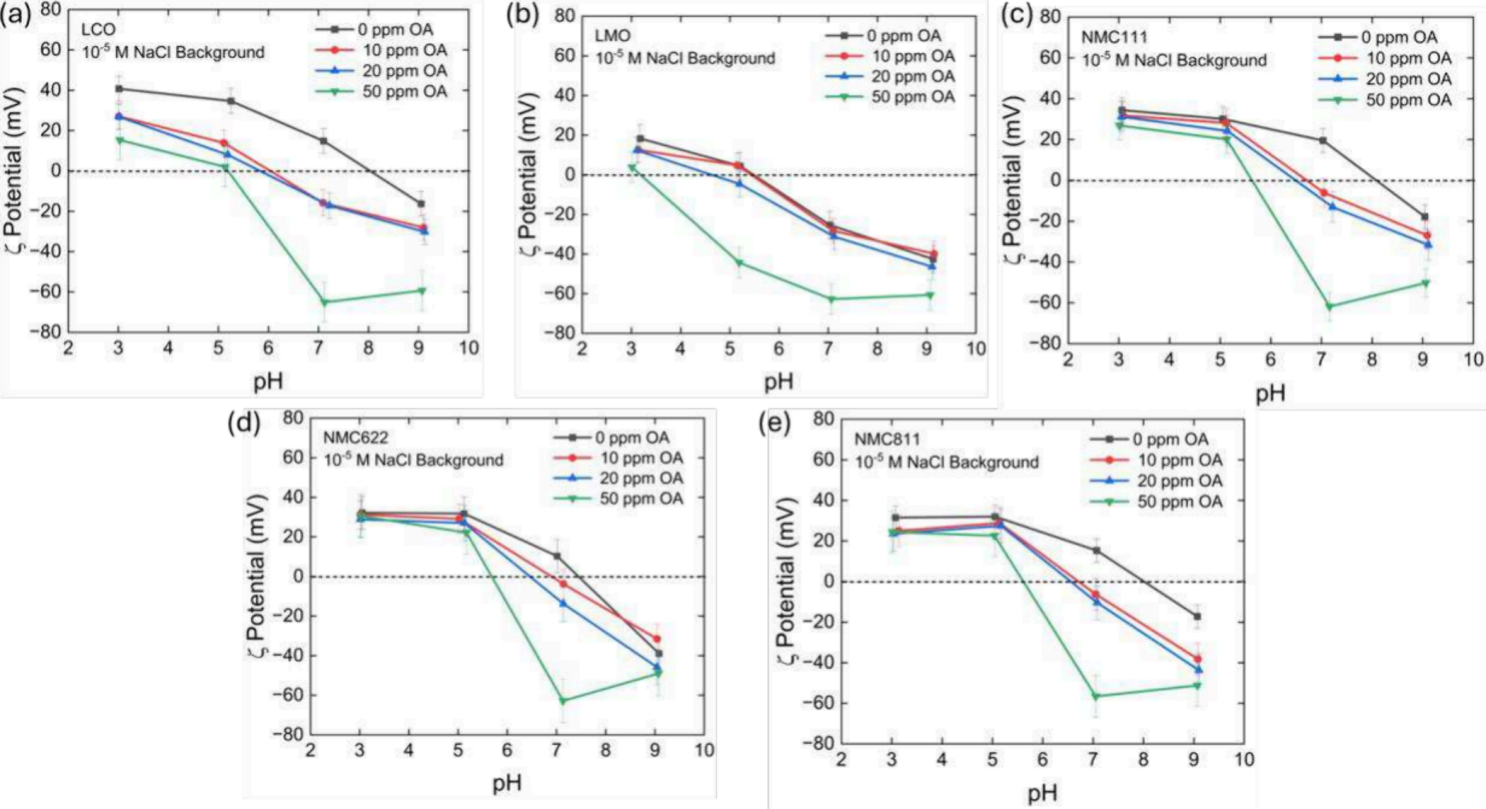
(a–e) ζ-potentials of LCO, LMO, NMC111, NMC622, and NMC811 as a function of pH in 10^–5^ M NaCl electrolyte with varying oleic acid concentration from 0 to 50 ppm oleic acid.

According to Figure 12, the point of zero charge (PZC) for NMC622 was 7.8, while NMC111, NMC811, and LCO had PZC values of approximately 8.2. Among all of the cathode materials investigated, LMO had the lowest PZC of 5.2. At pH 5, where the optimum separation occurred, NMC and LCO cathodes were positively charged, whereas LMO was neutrally charged.^39,56,57^ At this condition, oleate ions can selectively adsorb onto the surfaces of NMC and LCO materials predominantly via charge–charge interaction. The adsorption of oleic anions renders the NMC and LCO surfaces hydrophobic, while the LMO surface remained unmodified due to weak adsorption of oleate ions.

Note that the presence of oleic acid (OA) especially at 50 ppm causes the ζ-potentials of all cathodes to become more negative compared to those shown in the absence of oleic acid, suggesting that oleate species might adsorb onto cathode surfaces. Oleic acid exhibits pH-dependent speciation in water with a p*K*_a_ of approximately 5.02.^58^ At pH 5, about 50% of the oleic acid molecules exist as deprotonated oleate anions (RCOO^–^), while the remainder exists in uncharged oleic acid molecules (RCOOH).^45,59^ At pH 5, NMC-type cathodes and LCO, which are positively charged, attract oleate anions via electrostatic interactions, leading to strong physical adsorption with coadsorption of oleic acid molecule (RCOOH). However, at pH 5, LMO is neutrally charged, which reduces its interaction with oleate anions, resulting in low floatability. This may explain why NMC and LCO cathodes show strong flotation at acidic conditions (pH 5), while the LMO cathode remains nonfloated. Based on this finding, a schematic representation of oleic acid adsorption mechanism is proposed in Figure 13.

**Figure 13:**
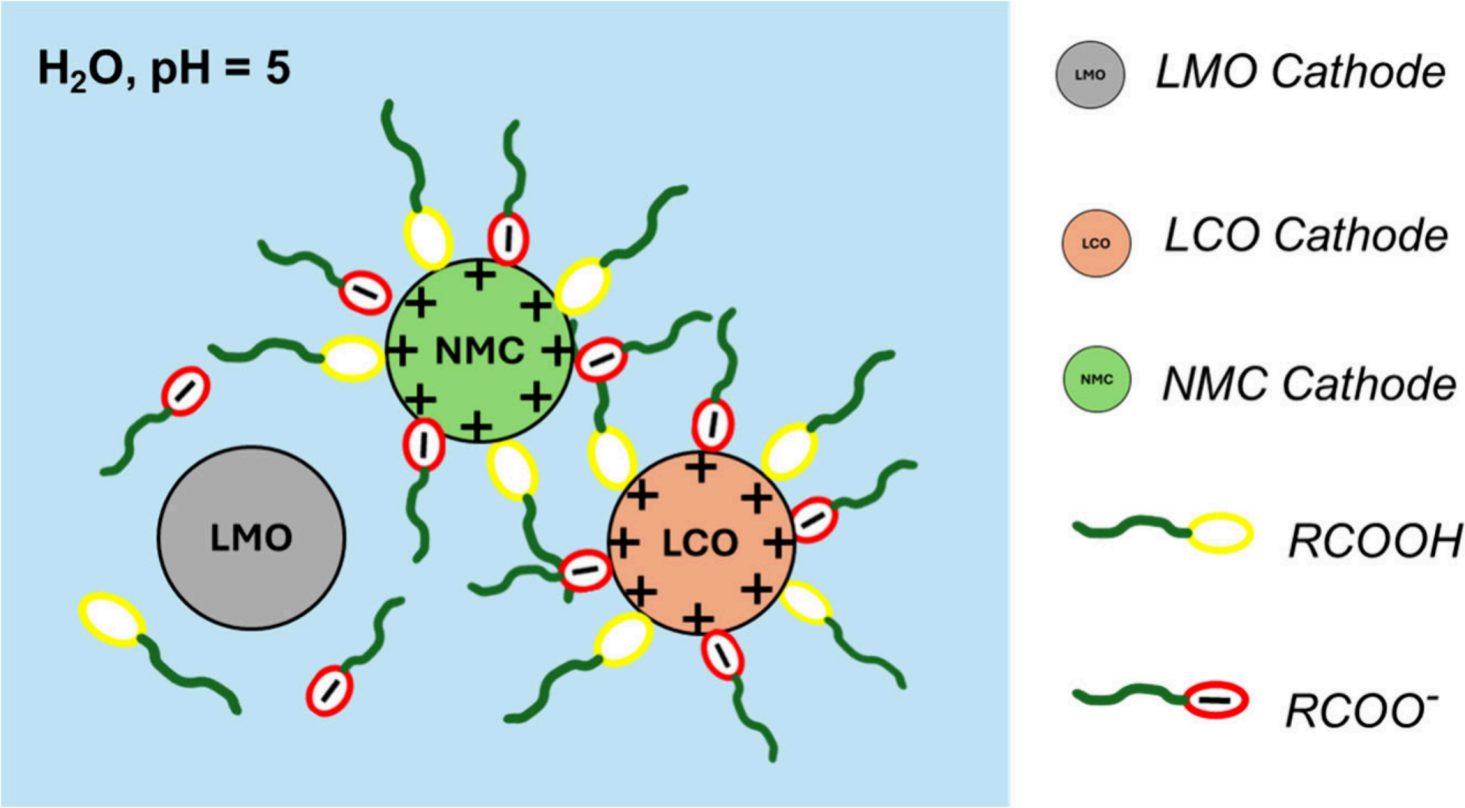
Schematic illustration of the adsorption of oleic acid on NMCs and LCO and LMO materials at pH 5.

Note also that the adsorption of oleic acid on LMO surfaces may occur at a higher OA concentration. This hypothesis is supported by the decrease in ζ-potential for LMO particles across the entire pH range investigated in this work. As shown in Figure 12b, at pH 5, ζ-potential of LMO particles dropped to nearly −43 mV with 50 ppm of OA. At lower OA concentrations, however, LMO ζ-potential remained the same. The high OA dosage may lead to partial flotation of LMO materials at pH 5, which has been shown in Figure 5b. This observation is consistent with the earlier results reported by Fuerstenau and Shiabata (1999),^45^ who found that manganese oxide minerals could float at the acidic conditions if the concentration of oleate is sufficiently high. In this scenario, flotation of manganese oxide is primarily driven by the weak physical adsorption of oleic acid molecules (RCOOH) onto the surface alongside the oleate anion (RCOO^–^).^45^ However, when the concentration of oleic acid used is low (<10^–5^ M), oleate anions will preferentially adsorb positively charged surfaces (e.g., NMC and LCO materials) instead of neutrally charged LMO surface.

Interestingly, the ζ-potentials of all cathode materials in the presence of 50 ppm of oleic acid between pH 7 and 9 are similar, around −60 mV, while their flotation performance differs significantly as shown in Figure 4. As pH increases from 7 to 9, LCO and NMC recoveries decline, while LMO recovery increases. This difference is likely due to the distinct hydrolyzed surface species formed at alkaline pH. Hydrolysis on LCO and NMC surfaces may suppress oleate adsorption, whereas hydrolysis on LMO may promote oleate adsorption due to the formation of MnOH^+^ cations. As has been reported in the literature, MnOH^+^ species are formed at pH 9 in the flotation of manganese oxide (MnO_2_), which promotes the chemisorption of oleate anions (RCOO^–^) and hence the flotation of MnO_2_.^45^ Similar chemisorption mechanisms of oleate anions have been also reported, where oleate anions adsorb onto metal oxide surfaces such as hematite, chromite, and rare earth oxide, by forming stable metal–oleate complexes.^40,42,44^ Further increasing pH to 10–11 suppresses the flotation of all cathode materials studied in this work, suggesting that excessive hydroxylation inhibits oleate adsorption. For LMO, the formation of manganese hydroxide Mn­(OH)_2_ at high pH restores surface hydrophilicity, reducing its floatability.

### 4.4 Summary

In summary, the flotation behavior of cathode materials is strongly pH-dependent and is governed by different adsorption mechanisms. LCO and NMC exhibit surface hydrophobicity at acidic to neutral pH (5–7) due to physisorption of oleate anions onto their positively charged surfaces, supplemented by adsorption of neutral oleic acid molecules. In contrast, LMO remains largely hydrophilic at low pH because its nearly neutral surface interacts weakly with oleate anions, allowing effective separation from LCO and NMC. At higher pH (∼9), MnOH^+^ species formed due to hydrolysis create positively charged surface sites, promoting oleate chemisorption and resulting in a peak flotation recovery for LMO. Further hydroxylation at pH > 10 suppresses adsorption and restores the hydrophilicity of LMO.

The proposed adsorption mechanisms of oleic acid onto these cathode materials are supported by flotation performance, contact angle measurements, and ζ-potential data. Nonetheless, additional spectroscopic investigations are needed to confirm and further elucidate these mechanisms, particularly the nature of oleic acid adsorption under alkaline conditions.

## 5 Conclusion

In the present work, a new flotation chemistry was developed to efficiently separate Ni-rich cathodes (NMC111, NMC622, NMC811) and lithium cobalt oxide (LCO) from lithium manganese oxide (LMO) using oleic acid as the collector. Laboratory-scale froth flotation results demonstrated that NMC-type cathodes and LCO exhibited strong floatability under acidic conditions (pH 5), whereas LMO remains in the pulp phase, enabling effective separation. Increasing the oleic acid dosage further enhanced the recovery of both LCO and NMC-type cathodes. However, separation efficiency declined under higher pH conditions due to increased floatability of LMO, which reduced process selectivity. An optimum separation efficiency of 85%–90% was achieved in the separation of NMC111/LMO, NMC622/LMO, NMC811/LMO, and LCO/LMO after one single stage.

The underlying mechanism was elucidated by using multiple complementary characterization techniques. Both the contact angle and bubble-particle attachment measurement revealed that NMC and LCO particles become hydrophobic following oleic acid adsorption, while LMO particles remained hydrophilic. ζ-potential and X-ray photoelectron (XPS) analyses confirmed that, under acidic pH range, oleate species preferentially adsorb onto the positively charged surfaces of Ni-rich and Co-rich cathodes through electrostatic adsorption of oleate anions and coadsorption of oleic acid molecules. In contrast, at higher pH, chemisorption dominates, and the hydrolysis of Mn^2+^ to MnOH^+^ on LMO surface enhances LMO floatability, thereby diminishing separation efficiency.

The separation efficiency was further validated using recycled LCO and NMC111 materials recovered from end-of-life Li-ion batteries. The present work highlights the utility of the froth flotation in separating mixed cathode materials from diverse feed materials. Moreover, the fundamental interfacial science study revealed, for the first time, the adsorption mechanism of oleic acids on a range of various cathode active materials used in Li-ion batteries.

## Supplementary Material



## Notes

Additional experimental results, including flotation separation results of NMC 622/LMO, NMC 811/LMO, and LCO/LMO and SEM and EDS mapping images of separated cathode material products (PDF)
