# Supplementary material for: Interfacial Chemistry Involved in Selective Separation of NMC/LMO and LCO/LMO Binary Cathode Materials by Froth Flotation Using Oleic Acid
Source: ACS Appl Mater Interfaces. 2026 Feb 24;18(9):14331–43. doi: 10.1021/acsami.5c19071 (PMC12983205; doi:10.1021/acsami.5c19071)
Supplement: Supplementary file 1 [file am5c19071_si_001.pdf]

Supporting Information

Interfacial Chemistry involved in Selective Separation of NMC/LMO and LCO/LMO Binary Cathode Materials by Froth Flotation Using Oleic Acid

Richard K. Oboh[1], Kaiwu Huang[2], and Seoung-Bum Son[3], Lei Pan[1,*]

[1] Department of Chemical Engineering, Michigan Technological University, Houghton, MI 49931, USA
[2]Department of Mining and Minerals Engineering, Virginia Tech, 445 Old Turner Street Blacksburg, VA 24061, United States
[3]Chemical Sciences and Engineering Division, Argonne National Laboratory, Lemont, Illinois 60439, United States

*Corresponding Author
Email: leipan@mtu.edu;
Phone: +1 (906) 487-2569

Supporting Data

Figures (S1a-b) to (S3a-b) show binary separation flotation results for NMC622/LMO, NMC811/LMO, and LCO/LMO, respectively. These tests were performed at pH 5.0 using oleic acid (OA) as a collector at varied dosages (200–400 g/t). As shown in Figures S1a, S2a, and S3a, NMC622, NMC811, and LCO showed high flotation recoveries. Increasing the OA dosage further improved flotation recoveries. At the highest dosage of 400 g/t, NMC622 and NMC811 both reached about 90% recovery within 3 minutes, while LCO achieved over 80% recovery. In contrast, Figures S1b, S2b, and S3b show that LMO recovery stayed below 20%, even with 400 g/t OA. These results suggest that oleic acid can selectively hydrophobized NMC-type and LCO cathode materials at pH 5.0, while leaving the LMO cathode materials hydrophilic. By controlling the pH and OA dosage, one can achieve efficient separation between NMC-type and LCO cathode materials and LMO cathode materials.

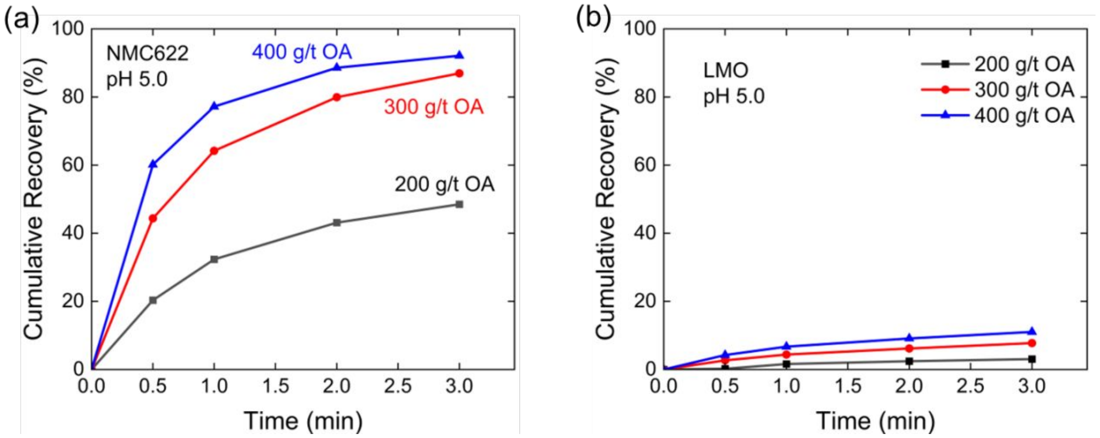

Figure S1. Cumulative recovery of NMC622 (a) and LMO (b) at pH 5.0 under varying oleic acid (OA) dosages (200, 300, and 400 g/t) in single-stage flotation.

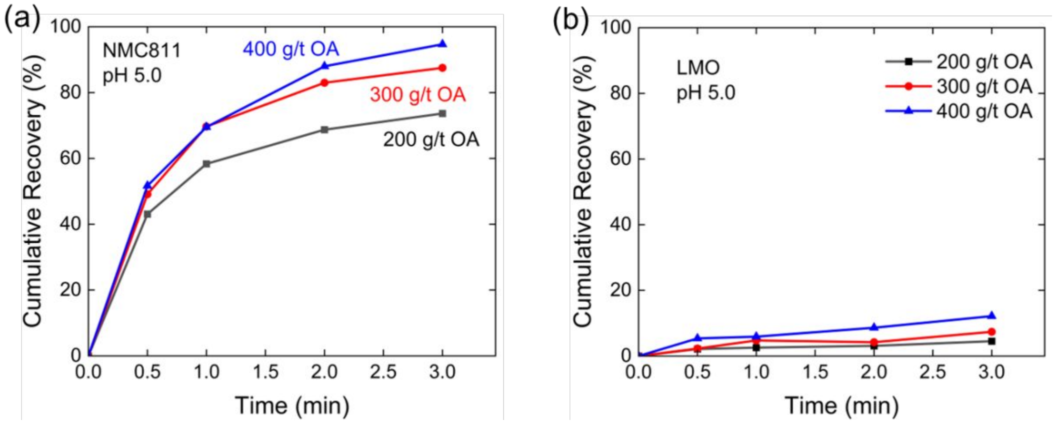

Figure S2. Cumulative recovery of NMC811 (a) and LMO (b) at pH 5.0 under varying oleic acid (OA) dosages (200, 300, and 400 g/t) in single-stage flotation.

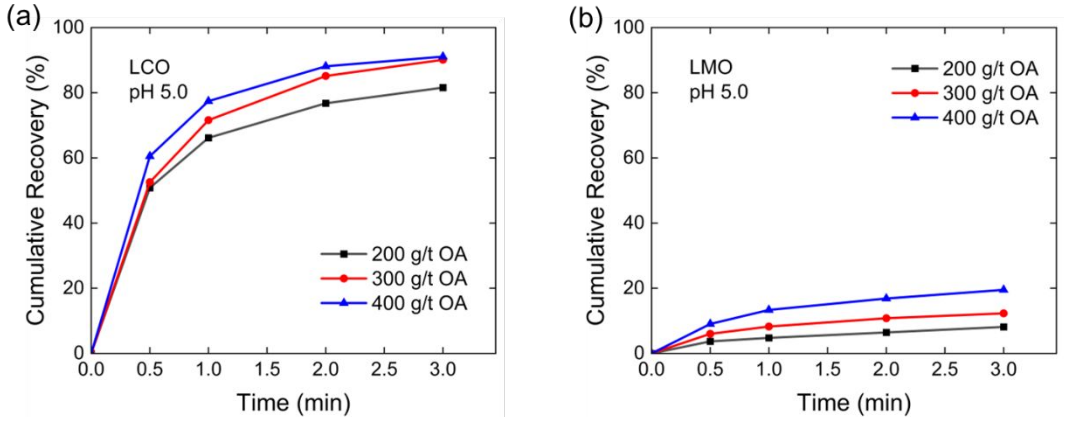

Figure S3. Cumulative recovery of LCO (a) and LMO (b) at pH 5.0 under varying oleic acid (OA) dosages (200, 300, and 400 g/t) in single-stage flotation.

Figure S4 shows the elemental distribution maps obtained with the froth and sink products in different binary separations, i.e., NMC622/LMO, NMC811/LMO, and LCO/LMO. These maps demonstrated the selective separation achieved in each system at pH 5.0 with optimal oleic acid (OA) dosages. The images of all froth products show high concentration of NMC or LCO particles, confirming their strong flotation behavior. The presence of Ni (green), Co (orange), and Mn (blue) in the froth products reinforces the selective recovery of NMC and LCO materials in the froth phase, leaving behind LMO in the pulp phase. The images of sink products, on the other hand, show an enrichment of Mn (blue), which is characteristic of LMO, confirming that LMO largely remained in the pulp phase due to its poor floatability at pH 5.0.

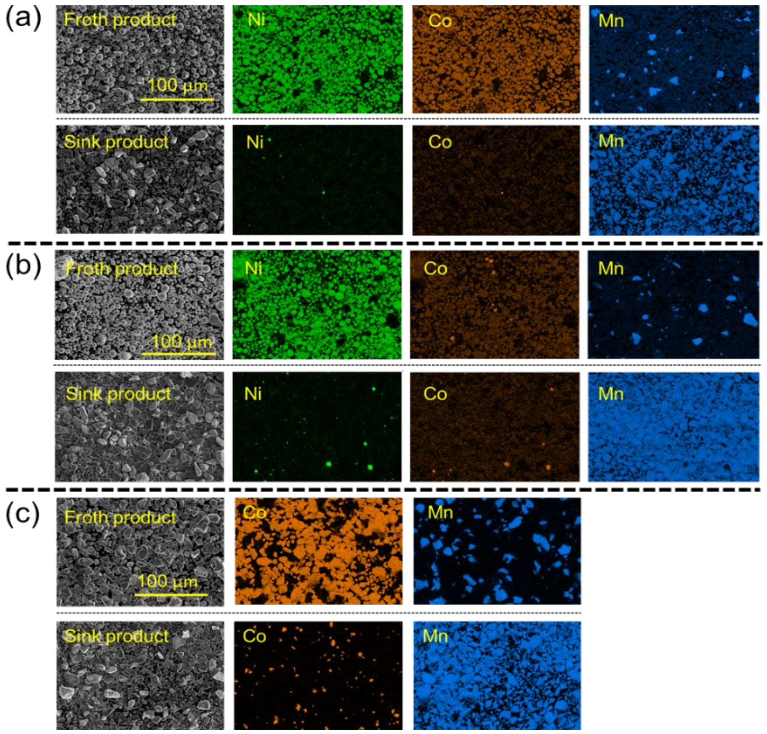

Figure S4. SEM/EDS elemental maps of the froth and sink products for a) NMC622/LMO, b) NMC811/LMO, and c) LCO/LMO separations at pH 5.0 using the optimal oleic acid dosage (400 g/t for NMC/LMO separations and 300 g/t for LCO/LMO separation).

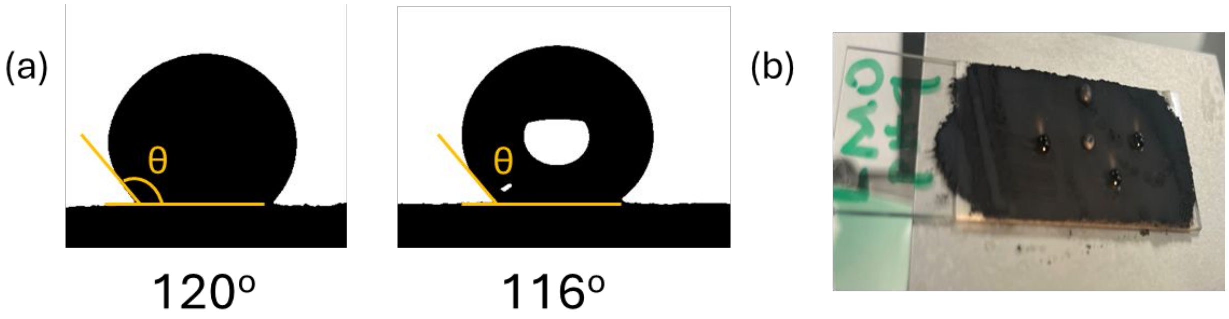

Figure S5: a. Images of water droplet on the LMO surfaces treated with oleic acid at pH 9. –b. A photo of LMO surfaces for contact angle measurement. The LMO particles were coated on a glass slice.

Figure S5 presents the contact angle measurements used to evaluate the wettability of LMO surfaces treated with oleic acid under alkaline conditions (pH 9). Figure(S5a) shows two representative water droplets deposited on oleate-conditioned LMO surfaces, where measured contact angles of 120° and 116° were obtained. The high contact angle values indicate that the LMO surface becomes strongly hydrophobic after conditioning with oleic acid at this pH. Figure (S5b) provides a photograph of the LMO-coated glass slide used for measurements, illustrating the uniform particle coating and the droplets placed for angle determination.
